# Investigating the potential of auxetic structures in tibial insert toward multi-range load-carrying knee implant

**DOI:** 10.1007/s00170-026-18816-w

**Published:** 2026-07-23

**Authors:** Ahmed ElZefzafy, Abdul Wasy Zia

**Affiliations:** Institute of Mechanical, Process and Energy Engineering (IMPEE), School of Engineering and Physical Sciences, Heriot-Watt University, Edinburgh, EH14 4AS UK

**Keywords:** Knee implant, Tibial insert, Auxetic structure, Finite element analysis, 3D printing, Instron 3367

## Abstract

The artificial tibial insert degrades within the human body over service time as a metallic femoral surface continues articulating against it. The degradation rate depends on gait patterns and mainly on the human body weight, as tibial inserts normally lack subject-specific customisation. This article investigates the feasibility of gyroid, square anti-chiral, and triangular auxetic structures for tibial insert applications. The mechanical performance of new tibial designs is compared against a conventional (bulk) tibial insert through computational and experimental studies. The preliminary stress-strain and deformation behaviours are studied with ANSYS Workbench. Whereas the Instron machine is used for experimental studies to test the 3D printed conventional and auxetic structure-based tibial inserts designed in this work. The experimental studies are performed under a wide range of quasi-static and cyclic loading conditions to understand their mechanistic performance and suitability for knee joint functionality. Among gyroid, square anti-chiral and triangular auxetic structures, the triangular design has demonstrated exceptional load-bearing capabilities up to 20 kN, an adequate deflection up to 2.3 mm, along with 34.8% reduction in component weight. The enhanced elastic recovery supported by auxetic structures can provide knee safety under impact loads. In addition, it is perceived that the auxetic structures-based tibial insert provides tunable effective stiffness, demonstrating the significance of auxetic lattice design and their suitability for a wider range of body weights to ensure mechanical stability, medical safety, and implant longevity.

## Introduction

**Fig. 1:**
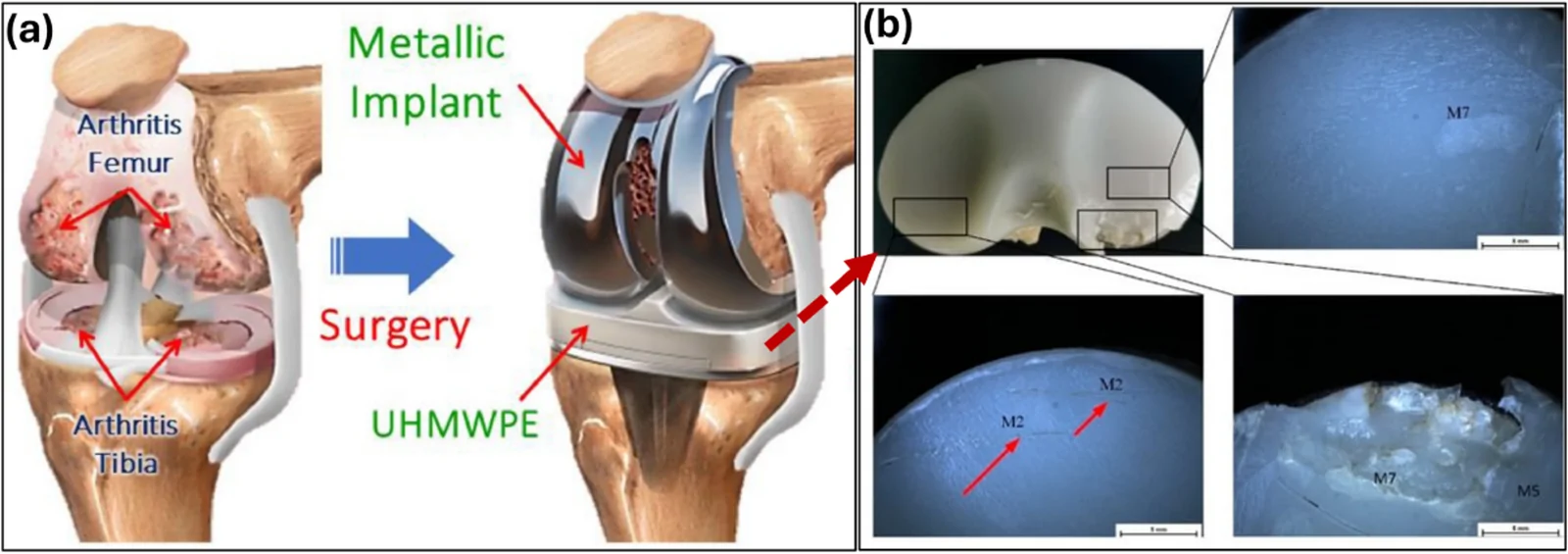
(**a**) Overview of the compromised human knee joint and restoration with Total Knee Arthroplasty. Adopted from [5]. Degradation of the tibial insert made of bulk plastic, over the service life. Adopted from [4]

Human knee joints may need to be replaced for several reasons, such as ageing, injury, and disease. Osteoarthritis [1] is a common disease leading to knee damage as presented in Fig. 1a (left image) and requires Total Knee Arthroplasty (TKA). TKA uses surgical procedures and application of artificial implants to restore or replace the human knee, depending on its condition, as shown in Fig. 1a (right image). Artificial knee joints are mainly composed of femoral and tibial components made of metal and attached to the thigh and shin bones, respectively. The tibial insert is situated between these two metal components, sitting on the tibial component, while the femoral component articulates against it [2]. The main function of tibial inserts is to emulate the natural cartilage of the knee. As such, they act like intermediate cushions that absorb shocks, distribute stresses, and facilitate the patient’s movement. The artificial joints are subject to mechanical degradation over time due to wear and biochemical material degradation. The material degradation will eventually lead to instability and imbalance during movement. The mismatch between the implant and bones may lead to refracture over time. The metal parts have been developed over time with the application of surface coatings to improve corrosion resistance, wear resistance, and hemocompatibility. However, major challenges still exist for the wear resistance of the tibial insert. The severity of such tibial insert wear is largely patient-specific, including such factors as physiology, body weight, and movement patterns. Significant numbers (millions) of plastic particles [3] released into human body over the life of tibial insert. Besides mechanical imbalance, these plastic particles reduce overall biocompatibility (cell viability) and accumulate in soft tissues and have the potential to act as tumour-initiating sites. Figure 1b gives an overview of the degradation of tibial insert and plastic deterioration from different locations during the service life [4].

Polymer-made tibial inserts have now been extensively used for several years for TKA applications. The literature [6] recommends controlling the material properties, cross-linking treatment, and contact mechanics to mitigate degradation. However, the wear of plastic for tibial insert is still a challenging problem. Figure 1b presents serious material degradation of the tibial insert for different cases, such as thin or thick poly insert, retrieved after 16 Y for a 71 Y old woman having a 100 Kg weight and retrieved after 18 Y for a 76 Y old man having a 120 Kg weight [7, 8]. Recent research studies and some commercial products are being reported on various strategies such as ultraviolet treatment [9], addition of antioxidants (such as vitamin E) [10], nanoparticles doped polymer composites [11] to improve the mechanical strength, wear resistance, and biological performance of ploy for TKA applications. However, the material degradation problem persists over time [12].

Beyond material science advancements, structural mechanics also holds good potential to help address this issue. As material degradation is linked to varying loading conditions and compression cycles, the application of auxetic structures may assist in uniform stress-strain distributions and energy balance during expansion and contraction in loading and unloading cycles. Auxetic structures have been around since the 1980’s [13], and their potential applications in various industries are evolving in recent years, such as running shoes, shape memory foam, and aeronautics [14]. Some human tissues, including tendons, skin, and arteries, have shown auxetic behaviour under loading conditions [15] and have been researched in tissue engineering applications, including bone scaffolding for enhanced biomechanical performance [14]. Considering the complex working conditions of the tibial insert, an auxetic structure could potentially offer a long-life and sustainable solution for TKR. However, the performance of the auxetic structure significantly depends on the type of lattice designs, such as re-entrant, chiral, and rotating structures, which are created by precise parameters and mathematical models, including Poisson’s ratio calculations [15]. Re-entrant structures are characterised by inward-facing angles that open during stretching and contract during compression. Chiral structures are known for their asymmetric and twisted shape, while rotating structures are composed of blocks connected in such a way that they pivot to amplify deformation. Furthermore, the exact geometry of auxetics is crucial, as even small changes in angles or lengths of parameters in the lattice plane configurations can disrupt the auxetic behaviour.

Ren et al. [16] conducted a holistic review of auxetic metamaterials and structures mentioning that 3D printing is a suitable fabrication method due to the great freedom it gives designers to create complex internal geometries; However, the high cost of manufacturing auxetic materials remains a challenge, hindering mass production capability. Yang et al. [17] compared the energy absorption performance of auxetic structures (re-entrant hexagon and arrowhead) with conventional non-auxetic honeycomb structures in a computational and experimental study and discovered that the auxetic geometries exhibited superior shock absorption, supporting their implementation in body protection pads to prevent injuries. Najafi et al. [13] demonstrated the energy absorption capabilities of Acrylonitrile Butadiene Styrene (ABS) 3D printed re-entrant, arrowhead, and anti-tetra chiral lattice designs, through quasi-static and low velocity impact tests. Their experimental studies have identified square anti-chiral with the best capability to absorb and dissipate energy under the application of external forces. The clinical translation may be useful for absorbing impacts during gait movements and leading to a less stiff, more comfortable and flexible knee implant. Shirzad et al. [14] have investigated the applications of Polylactic Acid (PLA) made 3D printed auxetic structures for biomedical applications, scaffolds in tissue engineering to enhance mechanical properties. Inspired by the natural structure of bone and tendon, they have created concave and convex bilayer architecture variants of a triangle auxetic unit cells and found more than 27% increase in both Young’s modulus and yield strength under compression. Thus, triangular unit cells may have the potential to reduce compress-related degradation if adopted for tibial insert application. Ziaie et al. [18] have conducted a numerical study to compare the performance of gyroid, diamond, and primitive structures for numerous properties like elastic modulus and yield strength in different Triply Periodic Minimal Surface (TPMS) arrangements. These structures are a type of metamaterial whose surfaces are derived from unique mathematical formulae and mimic the natural structure of various tissues in the body, including bone. Their study explored an inverse relationship between stiffness and porosity. The porous gyroid structure was discovered to have the lowest stiffness among the three lattice designs. Extrapolating an analogy, the smooth and isotropic geometry of the gyroid also closely resembles the natural scaffold of the tibiofemoral joint, proposing it as a potential lattice design for tibial insert application.

This article computationally and experimentally investigates the mechanistic behaviour of gyroid, square anti-chiral, and triangle lattice structures for tibial insert application. The finite element analysis is performed to understand stress concentrations, and experimental compression and fatigue tests are performed to assess the suitability of the auxetic tibial insert under a wide range of loading conditions. This study provides mechanistic insights by evaluating the performance of auxetic knee implant structures under uniaxial compression, which represents the predominant physiological loading condition experienced by knee implants. While this analysis focuses on axial compression, knee implants in vivo are exposed to complex multidirectional forces during daily activities. Therefore, this study validates the feasibility of auxetic structures for knee tibial insert application and develops a foundation to extend investigation for detailed experimental testing and laboratory-scale validation under multiaxial loading conditions for comprehensive assessment of implant performance. Such investigations could facilitate the optimization of auxetic implant designs, ultimately supporting the development of safer, more durable, and patient-specific knee replacement solutions.

## Methodology

The mechanistic performance of the auxetic tibial was assessed through computational and experimental studies.

### Computational studies

**Fig. 2:**
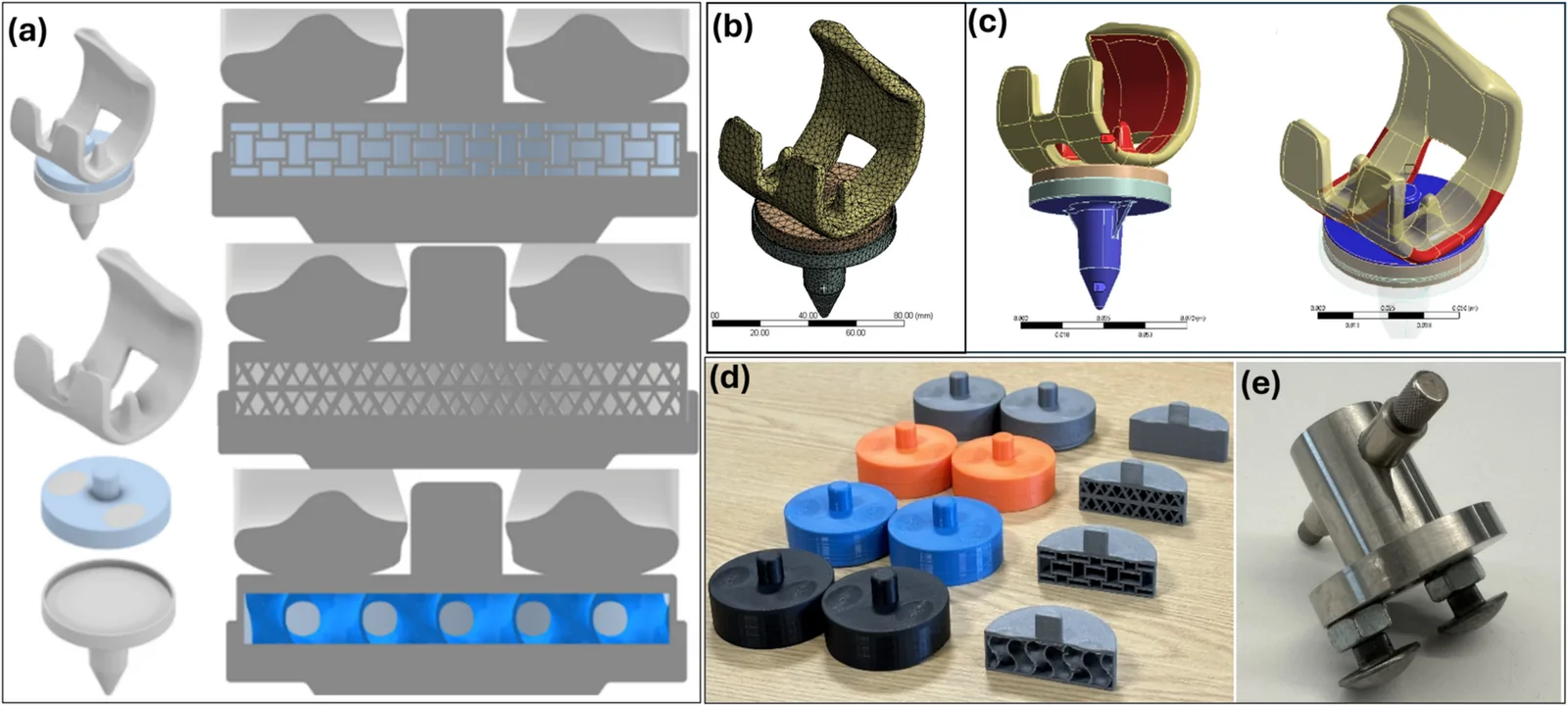
(**a**) 3D modelling of the knee joint and cross-sections of tibial inserts design with gyroid, square anti-chiral and triangular lattice structures. (**b**) Meshing and (**c**) boundary conditions for Finite Element studies. (**d**) Full and sectional 3D printed tibial inserts and (**e**) in-house design and fabrication of jig to mimic mechanical testing knee function

Referring to computational methodology, a basic 3D model of the knee implant was outsourced [19] and the tibial insert was redesigned in-house using Autodesk Inventor. Numerous locking mechanisms were considered and finalised, drawing inspiration from BS ISO 7207-1:2007 [20]. Three additional tibial inserts were designed having gyroid, square anti-chiral, and triangle lattice structures, along with a conventional tibial insert, as shown in Fig. 2a. The lattice design selection and dimensional aspects were based on the literature recommendation. The triangle auxetic unit cell design parameters were adapted from Shirzad et al. [14], which has shown enhanced Young’s modulus and yield strength increase over 27% under compression loading. Likewise, the choice of square anti-chiral geometry was adapted from Najafi et al. [13]. The gyroid structure was based on the study from Ziaie et al. [18]. A Finite Element Analysis (FEA) was performed in ANSYS Workbench 2024 as an intuitive decision tool for experimental studies. Table 1 presents the material properties assigned to metallic (femoral and tibial) components, which are usually made of titanium alloy, and to the tibial insert which is usually made of Ultra-high molecular weight polyethylene (UHMWPE).

**Table 1:** Mechanical and Material properties used for femoral and tibial components and tibia insert. Data adopted from [21, 22]

Mechanical and Material Properties	Tibial Insert	Femoral and Tibial Components
	UHMWPE	Ti6Al4V
Density (kg/m^3^)	945	4420
Young’s Modulus (MPa)	800	114,000
Poisson’s Ratio (unitless)	0.46	0.34
Tensile Yield Strength (MPa)	21	870
Tensile Ultimate Strength (MPa)	39	940

As part of FEA simulation, a mesh convergence test was conducted, and boundary conditions were assigned mimicking the knee anatomy. The mesh convergence ensures the sustainable computing powers require to solve the numerical simulations. A tetrahedral shape with a 2 mm element was perceived as an optimal element size, corresponding to 28,657 elements and 51,835 nodes, as shown in Fig. 2b. Referring to the boundary conditions, the bottom of the tibial component was fixed from the bottom to mimic the surgical procedure, as it is embedded in the shinbone. Whereas, the coefficient of friction between the femoral component and tibial insert was used as 0.2 [23], a common value for the friction coefficient between titanium and UHMWPE. Additionally, to minimise movement in other directions, bonded contact was used between the tibial insert and the tibial component slot. The boundary conditions are presented in Fig. 2c. Referring to the loading conditions, the knee joint experiences a variety of forces and moments. According to BS ISO 14243-1, the primary force is in the standing position along with the mechanical axis instead of the anatomical axis [24]. As such, a compressive axial force was applied to selected inner surface areas of the femoral component, as seen in Fig. 2c, to simulate body weight from the femur bone. Moreover, loads ranging from 50 to 100 kg were applied in increments of 10 kg, representing a wider range of loading conditions for the patients whose weight range from 100 to 200 kg distributing equally over two knees. The total deformation and von Mises stress were calculated to understand the mechanistic behaviour of tibial inserts.

### Experimental studies

Considering the FEA insights, the experimental studies were designed within resource limitations. PLA was used as a 3D printing filament, while the thickness of tibial insert used in FEA was doubled to meet the nozzle resolution. Fused Deposition Modelling (FDM) was used to print 3D models to understand the effect of auxetic structures on the mechanistic performance of tibial inserts. The CAD models were exported as STL mesh files and subsequently processed in Bambu Studio to generate the layered G-code toolpaths required for physical fabrication. The Bambu Lab P1P 0.4 mm nozzle, set to a layer height of 0.1 mm and 100% sparse infill density, was used to manufacture the tibial inserts as shown in Fig. 2d. Having the tibial inserts oriented facing up, the prints were achieved with no rafts or supports required. The nozzle temperature used was 220 °C ensuring optimal plastic flow, while the heated bed temperature was 65 °C to provide required adhesion to keep the print flat and avoid warping.

The mechanical testing was performed with an Instron 3367 ultimate tensile test machine. A customised jig was designed and fabricated in-house (Fig. 2e) to mimic the femoral bone contact with the tibial insert and simulate femoral loading. The bespoke steel piece was crafted to fit in the Instron universal testing machine via pin fastener and simulate axial loading similar to real anatomy and analogous to the FEA force boundary condition. This was achieved by the downwards loading from the two spherical-head M10 carriage bolts applied directly to the tibial insert dimples, mimicking the contact from the curved edges of the lateral and medial condyles (lowest parts of femoral bone).

The mechanical performance of auxetic structures was assessed through a quasi-static test, increasing the compressive force from 0 N to 30 kN, at a constant displacement rate of 30 mm/min, with data recorded every 100 ms. Whereas the second type was a cyclic loading test to assess the elastic recovery, where a maximum load of 2,600 N was applied during each cycle. This value was derived from the peak of the time-varying axial force from ISO 14243-1 [24]. Twenty cycles were conducted for each specimen at a 1,000 N/s loading rate.

## Results

**Fig. 3:**
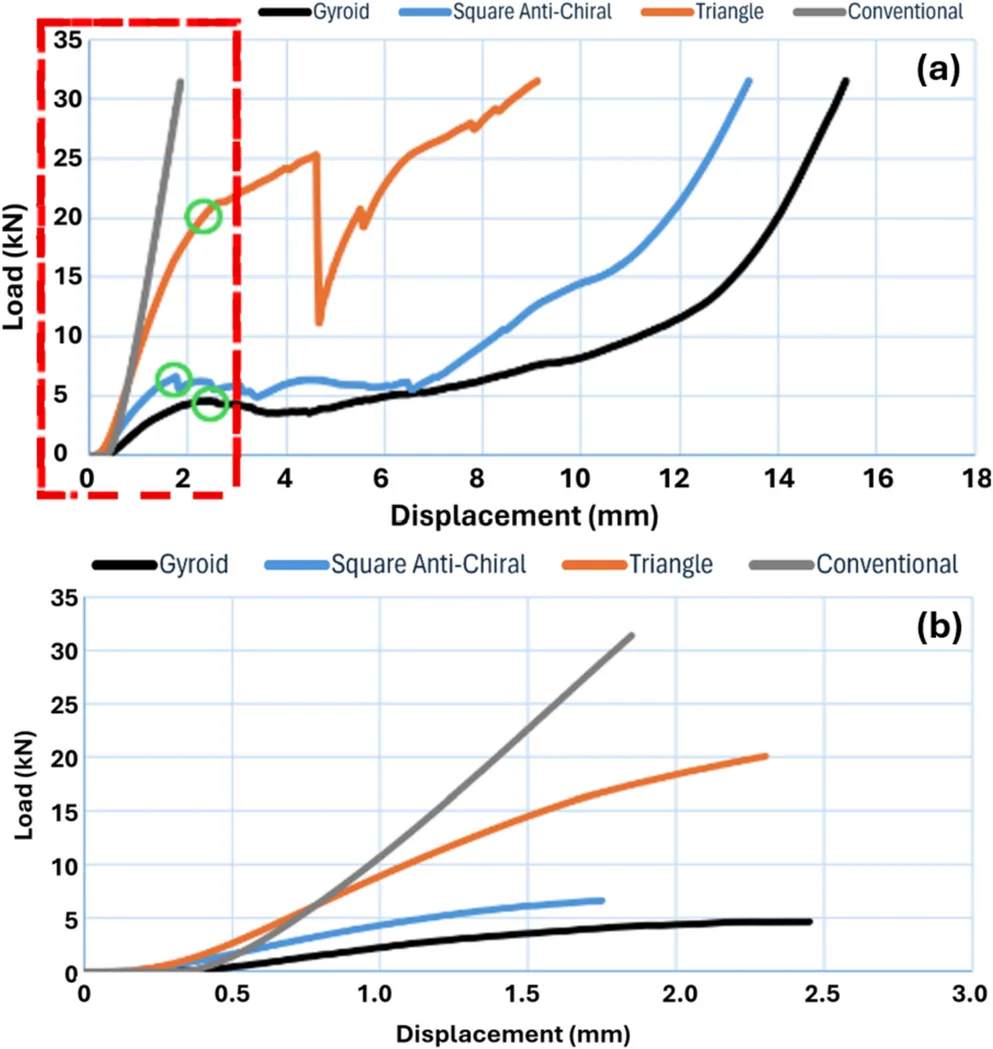
Experimental quasi-static load-displacement curves (**a**) up to the machine capacity of 30 kN and (**b**) load-displacement curves before the yielding point

Figure 3a gives the overview of the entire experimental quasi-static compression test for the tibial insert spacers, where the yield points of the specimen are circled in green, and an area of interest has been highlighted in a red rectangle. Most of the yield points were determined by the Instron machine automatically, however, for the triangle specimen a yield point was determined and selected manually. Figure 3b displays a close-up of the region highlighted in Fig. 3a.

Figure 3b includes the exact values of load and displacement that each auxetic structure yielded at. The conventional did not yield and pushed the machine until 31,384 N and 1.85 mm. The triangle auxetic yielded at 20,066 N and 2.30 mm, the square anti-chiral auxetic yielded at 6,625 N and 1.75 mm, and the gyroid structure yielded at 4,621 N and 2.45 mm. A summary of load and displacement values at yield points is presented in Table 2.

**Table 2:** Load and displacement values at the yield point for conventional and auxetic structure-based tibial inserts

Property	Conventional (Bulk)	Square Anti-Chiral Structure	Triangle Structure	Gyroid Structure
Load at yield (kN)	N/A (> 31.38)	6.63	20.06	4.62
Displacement at yield (mm)	N/A (> 1.85)	1.75	2.30	2.45
Mass (g)	63.5	29.0	41.4	24.5
Load at yield relative to mass (kN/g)	N/A (> 0.494)	0.229	0.484	0.189

**Fig. 4:**
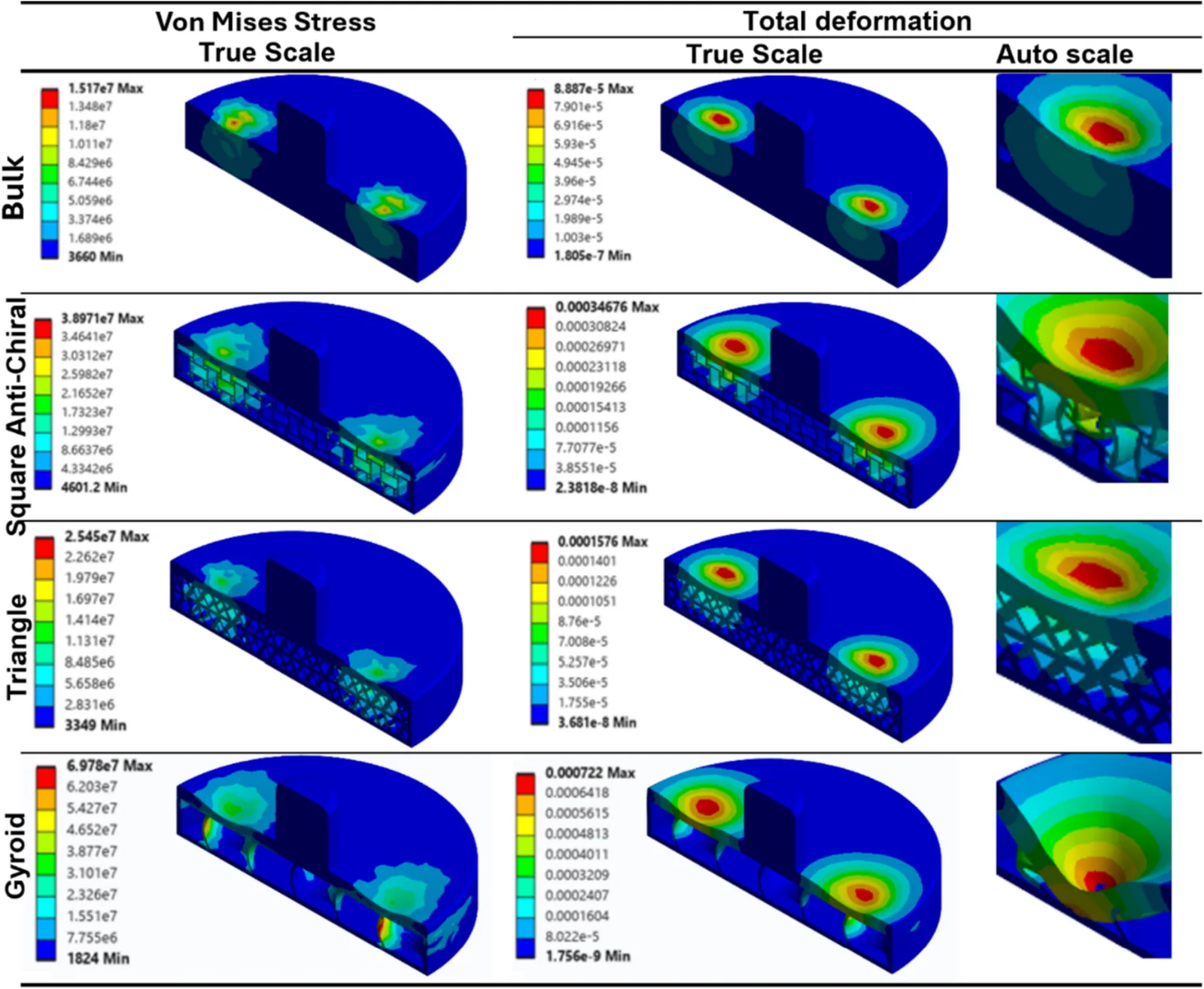
Von Mises stress distribution and maximum deformation at 100 kg in conventional and auxetic structures based tibial inserts

Figure 4 presents the von Mises stress distributions and total deformations in the tibial inserts when simulated under a 100 kg load. The maximum stress concentration of 69 GPa was observed for Gyroid structures, then 39 GPa for the square anti-chiral structure, 25 GPa for the triangle structure and a minimum of 15 GPa for the bulk tibial insert. The outcomes are logical considering contact mechanics as surface area per unit applied load reduces in the auxetic structures, therefore the stress increases proportionally. The deformation trends were also consistent with stress mapping. The exaggerated deformation views are also presented in Fig. 4 to reflect the deformation behaviour of lattice structures. It can be seen that the gyroid is most vulnerable and the triangular design is least vulnerable to deformation among all three structures.

**Fig. 5:**
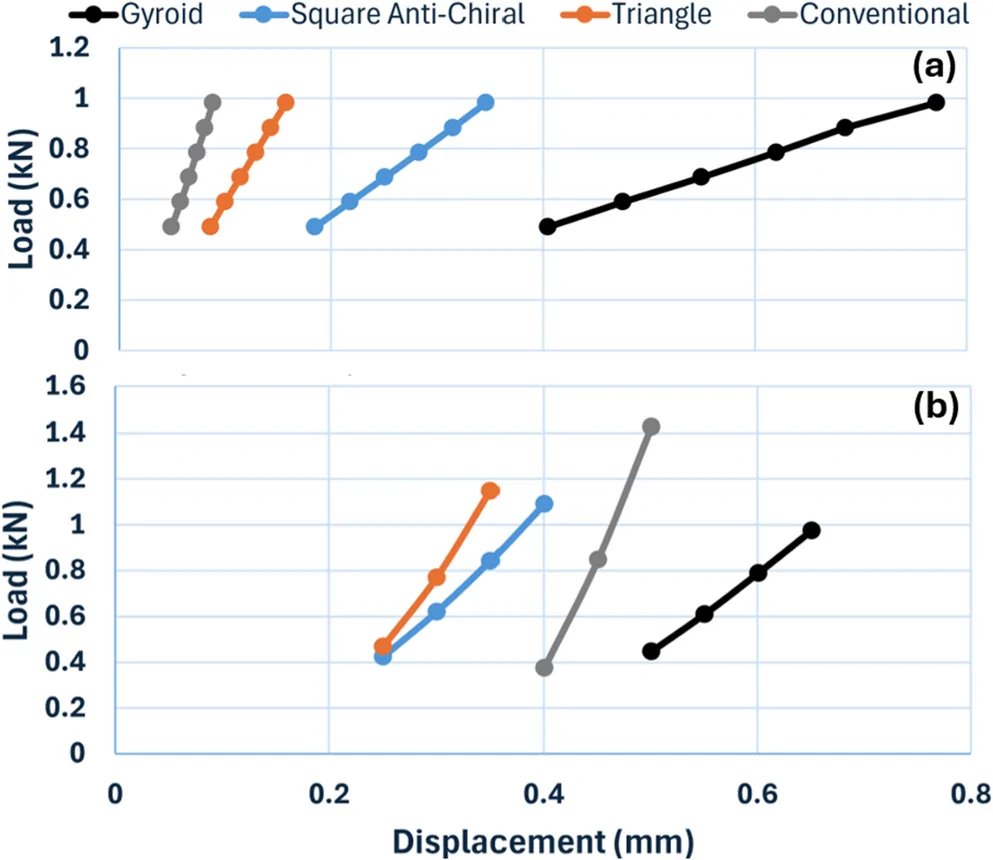
(**a**) Computational (UHMWPE, 10 mm thickness) and (**b**) experimental (PLA, 20 mm thickness) results of load vs. displacement of conventional and auxetic structures based tibial inserts

Figure 5a represents the load vs. displacement graph obtained from FEA, while Fig. 5b represents the load vs. displacement graph obtained from experimentation. Although multiple knee implant components were used in the FEA simulation for boundary conditions, the results represent the tibial insert data only.

**Fig. 6:**
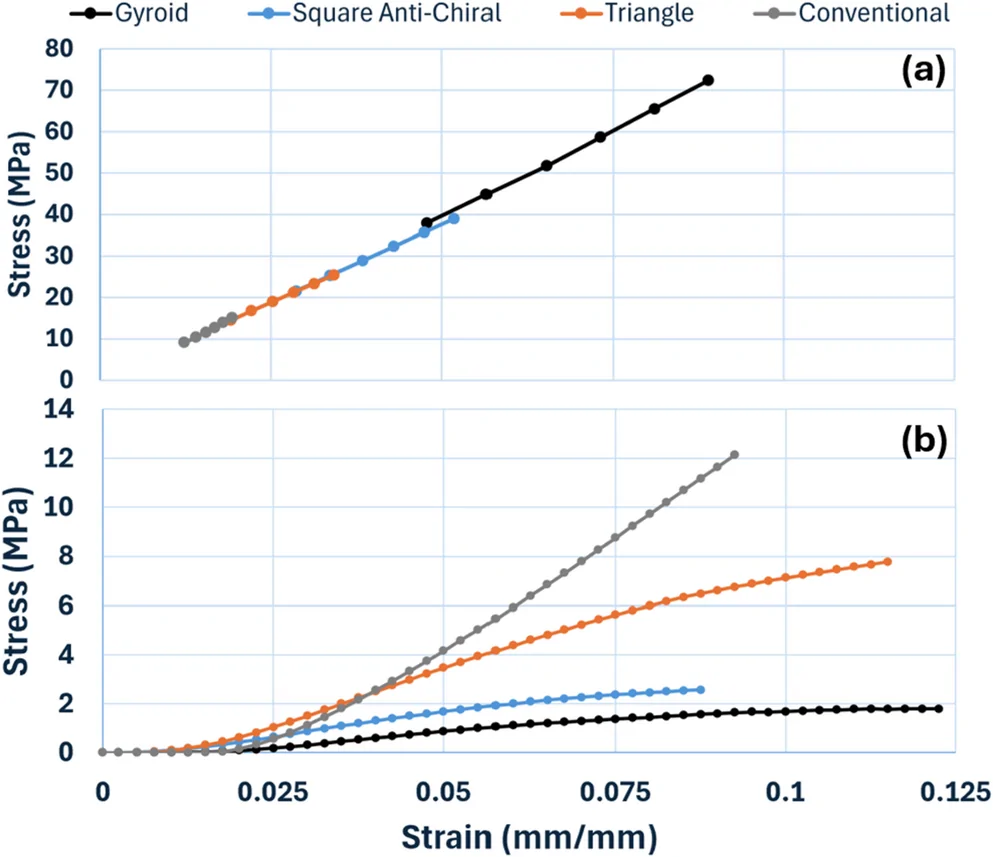
(**a**) Computational and (**b**) experimental results of stress-strain behaviour of conventional and auxetic structures based on tibial inserts

Figure 6a represents the stress vs. strain graph obtained from FEA, while Fig. 6b represents the stress vs. strain graph obtained from experimentation. For the FEA, the maximum von Mises stress and the maximum strain were recorded for each iteration. Whereas in the experiment test, the stress was automatically calculated by dividing the vertical load value by the initial cross-sectional area of the tibial insert, and the strain values were calculated by dividing the vertical deformation values by the initial height of the tibial insert.

**Fig. 7:**
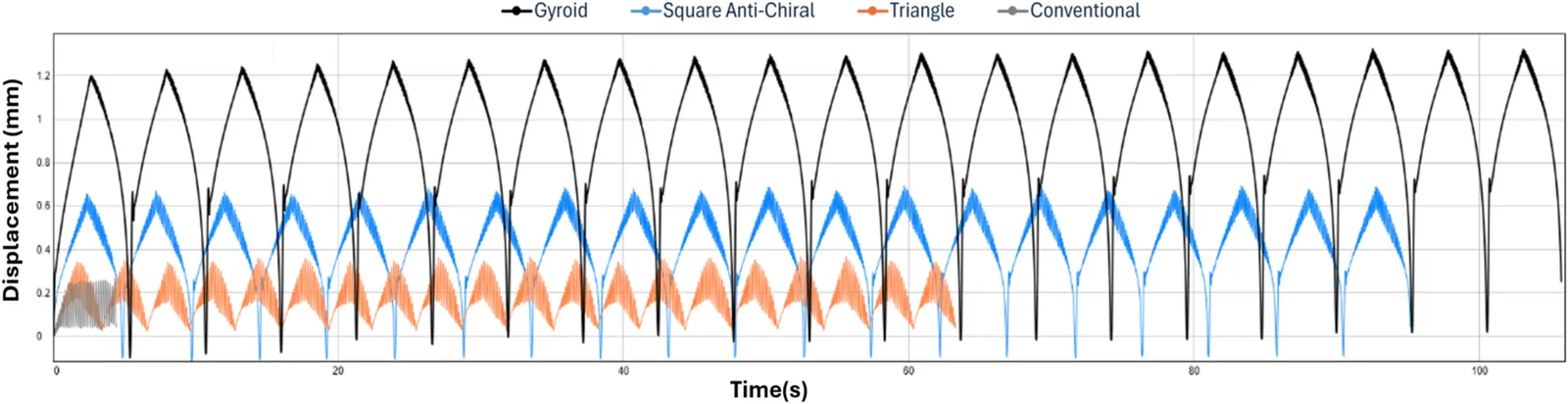
Experimental results of the cyclic loading test to assess the elastic recovery of conventional and auxetic structure-based tibial insert

Cyclic loading tests were performed to assess the elastic recovery of auxetic-based tibial inserts to understand their performance under impact loads and life expectancy. Figure 7 presents the displacement as a function of time and number of cycles. The gyroid had the largest peak-to-peak amplitude with around 1.3 mm, followed by the square anti-chiral with 0.55 mm, the triangle with 0.31 mm, and finally the conventional with 0.21 mm. Moreover, the difference in frequencies and wavelengths between specimens was notable. Following a similar trend as amplitudes, the gyroid presented the longest frequency and wavelength, then the square anti-chiral, then the triangle, and finally the conventional spacer.

## Discussion

The yield points in the load-displacement slopes seen in Fig. 3a represent the point of transition from elastic deformation to plastic deformation and can be identified when the linear trend ends, and the graph starts to curve. In a linear region, the material behaves elastically, meaning it returns to its original shape when the load is removed. Furthermore, this linear trend follows Hook’s Law, where stress is directly proportional to strain. Once the graph starts to curve, however, the material begins to yield and enters the plastic region, meaning permanent deformation occurred. The post-yield behaviour gives details on mechanistic behaviours. The increasing slope after yielding suggests a strain-hardening effect where the structure resists further deformation, as observed for the triangle structure. In contrast, the decreasing slope after yielding, as observed for the gyroid and square anti-chiral structures suggest that the material is undergoing softening and could indicate energy absorption capabilities. Further, the bumpy points observed in the auxetic structures may represent multiple yield points, corresponding to progressive collapse of auxetic lattices.

Although all the auxetics were eventually subject to the maximum 30 kN, only the conventional tibial insert was able to withstand it without yielding, as shown in Fig. 3b. The yield limits of the conventional tibial insert remain unknown due to machine capacity. However, it is worth noting that the raw PLA has standard stiffness, as all tibial inserts were made with the same filament. Therefore, apparent stiffness could be tuned to different values with the application of auxetic structures as reflected in their load-displacement curve in Fig. 3b. As such, in the linear elastic region, a steeper slope suggests a rigid structural design, while a shallower slope implies greater flexibility due to the auxetic geometry. From their slopes, the most rigid was the conventional, followed by the triangle, square anti-chiral, and finally the gyroid, which was the most flexible. A comparison of load endurance relative to component mass suggests the triangle structure had excellent load-bearing capabilities, especially a considerable reduction of mass up to 34.8% when compared to the conventional tibial insert. The large displacements before failure suggest that the auxetic structures can absorb significant energy and present deflection, making them a suitable choice for cushioning applications.

The behaviour of the gyroid structure in both simulation and experiment studies is very close, as seen in Fig. 5a and b. Not only is it the shallowest in terms of slope, but it also has the largest deformation/displacement values, in terms of magnitude, for the applied loads. The behaviour of the gyroid, square anti-chiral, and the triangle auxetics relative to one another is also similar between simulation and experiment. In both cases, the triangle structure is the most rigid, the gyroid is the most flexible, and the square anti-chiral is in the middle, as indicated by their respective load-displacement slopes. The displacement values of the square anti-chiral are also in a similar range in terms of magnitude, while the triangle had significantly lower displacement values in the simulation than in the experiments. The overall FEA simulation and experiment behaviour of tibial inserts are, although similar, but quantitative differences are associated with choice of material and properties such as UHMWPE in FEA and PLA in experimental studies, intrinsic material defects and process uniformity, and accuracy differences in computational and experimental boundary conditions, etc.

The other comparison between computational and experimental studies could be developed on a stress-strain basis, which presents the effective Young’s modulus. Effective Young’s modulus represents the overall stiffness of a structure, reflecting how much it resists deformation under applied stress due to material and geometry effects. Too stiff or ductile materials are perceived as not suitable for tibial insert applications, as high or low stiffness will present a brittle failure and unreasonable elongation, respectively. The tibial degradation will start in the early post-surgery months in both cases. Hence, there should be threshold stiffness compliant to tibial insert working environment. The calculation of the effective Young’s modulus value derived from the slope of the FEA stress vs. strain graph are presented in Fig. 6a and calculated using Eq. 1 [25] as follows:

1 $$\mathrm{E}\mathrm{e}\mathrm{f}\mathrm{f}=\frac{65-30\:\mathrm{M}\mathrm{P}\mathrm{a}}{0.08-0.04\:\mathrm{m}\mathrm{m}/\mathrm{m}\mathrm{m}}=875\:\mathrm{M}\mathrm{P}\mathrm{a}$$

This value of 875 MPa falls within the typical range of UHMWPE true Young’s modulus, which ranges between 700 MPa and 1 GPa. However, tunable effective Young’s modulus was observed in the experiment studies, where each specimen had a different slope as seen in the stress vs. strain graph in Fig. 6b. Approximating the effective Young’s moduli of various specimens from their slopes in Fig. 6b, the values come out to roughly 170 MPa for conventional, 85 MPa for tringle, 40 MPa for square anti-chiral, and 20 MPa for gyroid. Here, the role of auxetic lattice structures is dominant, irrespective of the 3D printing of all specimens made from the same filament. Along with lattice structure, material impurities and defects, printing temperatures, etc., also contribute to the effective Young’s modulus to some extent.

The conventional tibial insert is made of bulk material; It presented the least compression and elastic recovery during the cyclic loading test. The gyroid, square anti-chiral, and triangle, however, showed compressibility and elastic recovery under the same loading conditions. As seen in Fig. 7, the 2.6 kN applied cyclic load started to indent and slightly degrade the gyroid and square anti-chiral lattices, while the triangle and conventional lattices displayed fatigue resistance. In a comprehensive review of auxetics, Ren et al. [16] mentioned how the porosity of auxetic structures often leads to a trade-off between some desirable properties (like increased shear resistance, improved indentation resistance, enhanced energy absorption) and mechanical performance when compared to solid materials. This trade-off was observed in the conducted study as the square anti-chiral and gyroid which displayed the highest elastic recovery in the cyclic tests withstood the least loads before yielding in the quasi-static compression tests, yielding at 6,625 N and 4,621 N compared to the conventional which could have exceeded 31,384 N.

The quasi-static compression and cyclic loading tests present an intuitive recommendation on the potential adoptability of lattice design for a specific patient weight range. The tunable effective stiffness observed in the auxetic tibial insert could be a pathway for a personalised tibial insert to ensure superior mechanistic performance and product longevity. This work serves as a foundation for investigating the performance of auxetic structures under increasingly complex and physiologically relevant loading environments. This work will help to design the next stage of detailed experiment to validate the functionality of auxetic structure under complex conditions. Future studies will extend the current framework to encompass multiaxial loading conditions, incorporating combinations of tensile, compressive, shear, and torsional stresses that more accurately represent the mechanical demands experienced in vivo. Furthermore, the application of cyclic loading across multiple frequencies and amplitude ranges will enable a comprehensive assessment of the structure’s dynamic behaviour under repetitive mechanical excitation. Such testing is essential for evaluating the ability of auxetic architectures to withstand rapid and repeated stress cycles while maintaining their structural integrity and functional performance. These investigations will provide valuable insights into key mechanical characteristics, including energy absorption and dissipation mechanisms, elastic energy storage and release, fatigue life, damage accumulation, and wear resistance under variable loading regimes. By subjecting the structures to conditions that closely replicate the complex biomechanical environment of the human knee joint during activities such as walking, running, stair climbing, and sudden directional changes, a more realistic understanding of their long-term durability and functional reliability can be achieved. Ultimately, this research pathway will contribute to the development of advanced auxetic materials and structures capable of mimicking the adaptive mechanical response of native knee tissues, thereby enhancing their potential for use in orthopaedic implants, tissue-engineered scaffolds, and other biomedical applications where sustained performance under demanding cyclic loads is critical.

## Conclusion

The role of auxetic structures is expanding as they reconfigure engineering mechanics across various industrial sectors. The work explores the potential of auxetic structures for artificial tibial insert applications. The typical, bulk tibial insert is made of polymer and degrades over time. In addition, the high density increases component weight and the absence of elastic recovery, which raises discomfort in impacts and shocks.

- Tibial inserts were reconfigured with gyroid, square anti-chiral and triangular lattice structures in this work. A finite element study was performed to understand the stress-strain distribution and deflection of auxetic lattices for a tibial insert mimicking knee implant operation.
- 3D printed tibial inserts were tested with an Instron machine up to 30 kN under quasi-static compression and 2,600 N cyclic loading to assess yielding, strength and elastic recoveries. Different lattices have demonstrated different yield points, stiffness levels, and deflections.
- The triangle auxetic showed exceptional load-bearing capabilities (up to 20 kN), with 34.8% lower mass compared to the conventional, coupled with the higher deflection (2.3 mm) under quasi-static testing, as well as excellent fatigue resistance through cyclic testing.
- The gyroid and the square anti-chiral also showed energy absorption capabilities; however, a trade-off between high stiffness and malleable flexibility persists.
- The tunable effective stiffness observed in the specimen tested reflects the potential adaptability of specific types of auxetic structures for tibial insert design for a particular weight range of patients. This will enhance tibial insert compliance with patient anatomy, mechanical stability, and product longevity.

The present study provides valuable insights through a uniaxial analysis, considering that the dominant physiological load in knee implants acts with compression in the axial direction. In clinical applications, implants are subjected to more elaborate multidirectional loading conditions; Hence, future studies may further enhance the current findings through detailed experimental testing and lab-scale validation of the studied auxetic structures under complex multiaxial loading environments to more aptly mimic the knee operation.

## Data Availability

All the data is presented within this article.
